# Pilot study on esketamine response in treatment-resistant depression: impact of pharmacogenetic, clinical, and demographic variables

**DOI:** 10.3389/fphar.2026.1783538

**Published:** 2026-05-05

**Authors:** Michaela Krivosova, Matteo Marcatili, Gessica Guerrera, Ranieri Domenico Cornaggia, Federico Luigi Motta, Tommaso Paolini, Beatrice Gamba, Emanuela Giampieri, Francesca Bertola, Beatrice Benatti, Massimo Clerici

**Affiliations:** 1 Biomedical Centre Martin, Jessenius Faculty of Medicine in Martin, Comenius University Bratislava, Martin, Slovakia; 2 Department of Mental Health, Fondazione IRCSS San Gerardo dei Tintori, Monza, Italy; 3 School of Medicine and Surgery, University of Messina, Messina, Italy; 4 School of Medicine and Surgery, University of Milano-Bicocca, Milan, Italy; 5 Cytogenetics and Medical Genetics Unit, Centre for Disorders of Iron Metabolism, San Gerardo Hospital, Fondazione IRCSS San Gerardo dei Tintori, Monza, Italy; 6 Department of Biomedical and Clinical Sciences Luigi Sacco, Department of Psychiatry, ASST Fatebenefratelli-Sacco, CRC ‘Aldo Ravelli’ for Neurotechnology & Experimental Brain Therapeutics, University of Milan, Milan, Italy

**Keywords:** BDNF, CYP2B6, esketamine, OPRM1, pharmacogenetics, TRD, treatment-resistant depression, psychotherapy

## Abstract

**Introduction:**

Esketamine, the S-enantiomer of ketamine, is a rapid-acting antidepressant approved for treatment-resistant depression (TRD) when used in combination with an oral antidepressant. Identifying reliable clinical or genetic predictors of treatment response remains a critical unmet need. This study aimed to evaluate the impact of selected clinical and genetic variables on esketamine response in a real-world TRD cohort.

**Methods:**

Thirty-two TRD patients received intranasal esketamine over 2 months (12 administrations) and underwent pharmacogenetic testing. Depressive symptoms were assessed at baseline and at each session. Response and remission rates were analyzed in relation to clinical, demographic, and genetic variables, including *BDNF* (rs6265), *OPRM1* (rs1799971) polymorphisms, and CYP2B6, CYP2C9, and CYP3A4 metabolizer status.

**Results:**

No single demographic, clinical, or genetic variable reliably predicted treatment response. Adjunctive psychotherapy emerged as the only factor significantly associated with remission. Because most patients reached the standard 84 mg dose under the protocol, nominal dosing explained little of the observed variability in outcomes. Exploratory analyses suggested that metabolic phenotype and concomitant pharmacotherapy may contribute to inter-individual differences in treatment response.

**Discussion and Conclusions:**

In real-world TRD care, variability in esketamine response appears to be driven less by patient selection or nominal dose and more by a combination of pharmacologic exposure, biological factors, and psychotherapeutic engagement. These findings support a multidimensional, clinically oriented approach to treatment optimization rather than reliance on a single predictor. Given the limited sample size, the study may have been underpowered to detect modest associations, and the results should be therefore considered exploratory. Future research should prioritize the co-optimization of dosing strategies and psychotherapeutic engagement in routine care, and confirm these findings in larger, prospective studies.

## Introduction

According to the European Medicines Agency, treatment-resistant depression (TRD)—previously defined as failure to respond to at least two different first-line antidepressants administered at adequate dose and duration with confirmed adherence—can now be considered even in patients who fail to respond to a single antidepressant given at the maximum tolerated dose and for an adequate duration (Butlen-Ducuing et al., 2024). The prevalence of TRD in real world clinical settings ranges from 6% to 55% (McIntyre et al., 2023). Management of TRD includes switching antidepressants or their combination, augmentation strategies with different pharmacologic agents, neurostimulation techniques such as electroconvulsive therapy (ECT) or repetitive transcranial magnetic stimulation (rTMS), and the use of rapid-acting antidepressants like ketamine and its S-enantiomer, esketamine (McIntyre et al., 2023). Esketamine has been approved as an antidepressant used in TRD in a combination with oral antidepressant and for the rapid reduction of depressive symptoms in psychiatric emergencies (Canady, 2020). It acts as a non-competitive antagonist of N-methyl-D-aspartate (NMDA) receptor, which subsequently leads to α-amino-3-hydroxy-5-methyl-4-isoxazole propionate (AMPA) receptor activation. A key component of its mechanism involves upregulation of brain-derived neurotrophic factor (BDNF) and its receptor, tropomyosin receptor kinase B (TrkB) (Vasiliu, 2023). Compared to racemic ketamine, the enantiomeric S-ketamine is four-fold more potent for the NMDA receptor, which enables administration of much lower doses and thus reduction of dose-dependent dissociative adverse effects (Bahji et al., 2021). Intranasal application, avoiding first-pass metabolism (FPM), allows a rapid systemic drug availability. In real-world clinical settings, approximately 54% of intranasal esketamine is absorbed directly into the systemic circulation via the nasal mucosa, while the remaining 46% is swallowed and undergoes intestinal and hepatic first-pass metabolism. Of this swallowed fraction, only a small proportion reaches systemic circulation (Perez-Ruixo et al., 2021).

**FIGURE 1:**
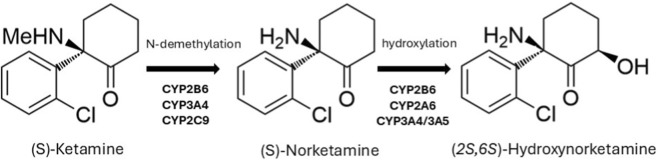
Simplified metabolism of esketamine.

Ketamine and esketamine are metabolized by CYP enzymes including isoforms 2B6, 3A4, 2C9, and 2A6 as shown in Figure 1 (Langmia et al., 2022). CYP2B6 is one of the most important enzymes involved in N-demethylation to the active metabolite norketamine. Variability in the activity of this enzyme could result in differences in ketamine metabolism (Wang et al., 2018): patients with wildtype genotype CYP2B6*1/*1 may exhibit up to a six-fold higher clearance compared to those with CYP2B6*6/*6 genotype (Li et al., 2013). *In vitro* data suggest a descending order of clearance efficiency across alleles: *CYP2B6* ^*^ *1* (wildtype*) > CYP2B6* ^*^ *4 > CYP2B6* ^*^ *26, CYP2B6* ^*^ *19, CYP2B6* ^*^ *17, CYP2B6* ^*^ *6 > CYP2B6* ^*^ *5, CYP2B6* ^*^ *7 > CYP2B6* ^*^ *9* (Wang et al., 2018). Reduced clearance may increase the risk of adverse drug reactions (ADRs). In conditions where CYP2B6 activity is insufficient, CYP3A4 and to a lesser extent CYP2C9 may take over the action of biotransformation to norketamine. Considering CYP2C9 isoform, it plays a minor role in biotransformation of ketamine. However, alleles *2 and *3 showed reduced catalytic activity, 26% and 3% of the wildtype variant, respectively (Zheng et al., 2017). Thus, genotyping of *CYP2B6, CYP3A4* and other isoforms involved could address the patients‘ individual needs and minimize ADRs.

Regarding the variability in the pharmacodynamics, mutations in the *BDNF* gene have been shown to affect the antidepressant effect (Liu et al., 2012). The most studied single nucleotide polymorphism (SNP) in this context is *Val66Met* (rs6265). Several animal and human studies suggest that *Val* allele might enhance the antidepressant effect of ketamine (Chen et al., 2019; Laje et al., 2012; Liu et al., 2012). Co-administration of other drugs acting on the NMDA receptor (lamotrigine, memantine, clozapine) could potentially cause pharmacodynamic interactions, but the clinical relevance remains to be investigated (Langmia et al., 2022).

Furthermore, the antidepressant effect of (es)ketamine might be influenced by individual dose, frequency, route of administration, age, sex, and other factors. Sex-specific differences might be dependent on hormonal levels. A recent analysis of controlled randomized trials showed that although the treatment efficacy did not differ between males and females, the rates of ADRs were higher in females, possibly due to induction of CYP enzymes by female reproductive hormones (Jones et al., 2022). Ethnicity might also play a role in the variability of esketamine response considering the various frequencies of alleles across populations.

Current literature on the genetics of esketamine treatment is limited (Franklin et al., 2025), with some studies analysing associations of *BDNF* (Beanes et al., 2022) and *OPRM1* polymorphisms (Saad et al., 2020) with esketamine response. Based on existing evidence suggesting the role of the BDNF pathway in esketamine’s mechanism of action and the impact of CYP450 enzymes on its metabolism, we hypothesized that genetic variability in *BDNF* and key metabolizing enzymes (*e.g., CYP2B6*) would be associated with treatment outcomes. Therefore, the primary aim of this retrospective, exploratory analysis was to explore the impact of these pharmacogenetic markers, along with clinical and demographic variables, on esketamine response in a real-world sample of TRD patients.

## Materials and methods

### Study participants

This retrospective study included 37 adult patients diagnosed with TRD, treated at the *Vademecum Fa.* Re (Unit for treatment resistant psychiatric disorders) at IRCCS San Gerardo dei Tintori di Monza, Italy, between November 2020 and September 2024. Patients were referred by their outpatient psychiatrists following non-response to at least two adequate antidepressant trials. The inclusion criteria were as follows: patients ≥18 years old, TRD diagnosis, major depressive disorder (MDD) or bipolar depression as a primary diagnosis, were clinically eligible for intranasal esketamine according to European Medicines Agency (EMA) product information (EMA, 2019), had completed baseline clinical assessment and available longitudinal psychometric data. The exclusion criteria were primary psychiatric diagnoses outside TRD indications (e.g., schizophrenia spectrum disorders, autism spectrum disorder), medical contraindications to esketamine (EMA, 2019), patients lacked sufficient clinical/psychometric data for analysis. After confirming the TRD diagnosis and verifying eligibility criteria, intranasal esketamine treatment was initiated according to unit’s the protocol. The treatment schedule consisted of an induction phase with administration twice weekly for first 4 weeks, followed by a maintenance phase with weekly administrations for the next 4 weeks. From week 9 onward, dosing frequency was individualized to either once weekly or every 2 weeks, depending on clinical response (EMA, 2019).

### Clinical assessment

At the initial consultation, comprehensive clinical data were collected, including personal, psychiatric, medical, and family history, comorbidities, and current symptomatology. To assess potential drug-drug interactions, the patients’ medication lists were reviewed for known inhibitors or inducers of CYP450 enzymes.

Symptom severity was evaluated using standardized psychiatric rating scales: the Montgomery–Åsberg Depression Rating Scale (MADRS) and the Hamilton Depression Rating Scale (HAM-D) for depressive symptoms, the Hamilton Anxiety Rating Scale (HAM-A) for anxiety, and the Brief Psychiatric Rating Scale (BPRS) for general psychopathology. These scales were administered at baseline and at each esketamine administration visit to monitor clinical response over time. Adverse effects were systematically monitored, and blood pressure was measured repeatedly in accordance with the treatment protocol.

The **MADRS** is a clinician-rated scale assessing ten dimensions of depression: apparent sadness, reported sadness, inner tension, reduced sleep, reduced appetite, concentration difficulties, lassitude, inability to feel, pessimistic thoughts, and suicidal ideation. Each item is scored from 0 (not present) to 6 (severe), yielding a total score ranging from 0 to 60. Scores of 0–6 indicate absence of symptoms, 7–19 mild depression, 20–34 moderate depression, and ≥35 severe depression. Treatment response is defined as a ≥50% reduction in total MADRS score from baseline, while remission is defined as a score ≤10 (Montgomery and Asberg, 1979; Quilty et al., 2013).

The **HAM-D**, widely used for decades, includes 17 items scored from 0 to 4. Total scores of 0–7 indicate no depression, 8–16 mild, 17–23 moderate, and ≥24 severe depression. Remission is defined as a score ≤7 (Hamilton, 1960; Ma et al., 2021).

The **HAM-A** assesses both somatic and psychological symptoms of anxiety using 14 items, each rated from 0 (not present) to 4 (severe). Items include anxious mood, tension, fears, depressed mood, and somatic symptoms across cardiovascular, sensory, respiratory, gastrointestinal, genitourinary, and autonomic domains. Scores <17 indicate mild anxiety, 18–24 mild to moderate, and 25–30 moderate to severe anxiety (Thompson, 2015).

The **BPRS** is a general psychopathology scale comprising 18 items rated from 1 (not present) to 7 (extremely severe). It evaluates a broad range of psychiatric symptoms including depression, anxiety, and psychosis (Thompson et al., 1994).

### Pharmacogenetic testing

Venous blood samples were collected in EDTA-containing tubes and used for DNA extraction to analyze pharmacogenetic polymorphisms. Variant analysis was performed using the commercial **Ion AmpliSeq Pharmacogenomics Research Panel** (Thermo Fisher Scientific, United States) in combination with the **Ion Torrent Genexus System** (Thermo Fisher Scientific, United States). This platform enabled efficient, high-throughput identification of common genetic variants associated with drug metabolism and response.

The following variants were analyzed: ***OPRM1*** *:* rs1799971 (c.118A>G); ***CYP3A4*** : *2 (rs55785340), *3 (rs4986910), *6 (rs4646438), *12 (rs12721629), *13 (rs4986909), *15 (rs4986907), *17 (rs4987161), *20 (rs67666821), *22 (rs35599367); ***CYP2C9*** : *2 (rs1799853), *3 (rs1057910), *4 (rs56165452), *5 (rs28371686), *6 (rs9332131), *7 (rs67807361), *8 (rs7900194), *9 (rs2256871), *10 (rs9332130), *11 (rs28371685), *13 (rs72558187), *15 (rs72558190), *16 (rs72558192), *27 (rs7900194); ***CYP2B6*** : *4 (rs2279343), *5 (rs3211371), *9 (rs3745274), *11 (rs35303484), *18 (rs28399499), *28 (rs34097093).

Allele function assignments and diplotype-to-phenotype classifications (i.e., metabolizer status) of *CYP3A4, CYP2C9* and *CYP2B6* were determined according to reference data from ClinPGx and CPIC guidelines (ClinPGx, 2025; Guidelines–CPIC, 2025).

In addition, the ***BDNF Val66Met* polymorphism** (rs6265, c.196G>A) was genotyped using polymerase chain reaction (PCR) followed by Sanger sequencing. The primers used were: forward 5′-ACA​AGG​TGG​CTT​GGC​CTA​C-3′, reverse 5′-AGA​GGA​GGC​TCC​AAA​GGC​A-3′, with an annealing temperature of 62 °C.

### Statistical analysis

Statistical analyses were performed using **GraphPad Prism 8.0.1** (GraphPad Software, San Diego, CA, United States) and **IBM SPSS Statistics v29** (IBM Corp., Armonk, NY, United States). Descriptive statistics were used to summarize sociodemographic and clinical characteristics.

Changes in symptom severity during esketamine treatment were assessed using **paired t-tests** for normally distributed data and **Wilcoxon signed-rank tests** for non-parametric data.

To evaluate the interactions between time points and metabolizer status, a **two-way repeated measures general linear model** was applied, with time as the within-subject factor and metabolizer type as the between-subject factor. **Mauchly’s test of sphericity** was applied for sphericity assumption, that is that variances of the differences between all possible pairs of within-subject conditions are equal. If it was significant (p < 0.05), **Greenhouse-Geisser correction** was used to reduce the risk of Type I errors.

The correlation between genotypes and various categorical characteristics was evaluated by **Chi-square (χ** ^**2**^ **) test**, while **Spearman’s rank correlation** was used for continuous variables.

**Hardy-Weinberg equilibrium (HWE)** was assessed by comparing observed and expected genotype frequencies using the Chi-square test in GraphPad Prism, with reference allele frequencies obtained from dbSNP for the European population (NCBI, 2025).

Overall, a p-value <0.05 was considered statistically significant. However, given the exploratory nature of this pilot study and the number of statistical tests performed, results should be considered preliminary and interpreted with caution. No formal correction for multiple comparisons was applied; findings with p-value approaching 0.05 were considered suggestive and interpreted conservatively.

## Results

Out of 37 patients initially included, one patient died due to an organic condition unrelated to the psychiatric disorder, two discontinued treatments for personal reasons and two did not undergo pharmacogenomic testing. Therefore, the final analysis was conducted on 32 patients. Demographic characteristics are presented in Table 1, clinical details related to psychiatric diagnoses in Table 2, and esketamine treatment parameters in Table 3.

**TABLE 1:** Socio-demographic data of study participants.

	N	%
Gender		
Male	13	40,6%
Female	19	59,4%
Age		
Mean ± SD	57.6 ± 13.8	
Min	20	
Max	79	
Educational level		
Middle school certificate	3	9.4%
High school diploma	17	53.1%
University degree	12	37.5%
Marital status		
Single	7	21.9%
Married	21	65.6%
Divorced	4	12.5%
Occupation		
Employed	6	18.8%
Unemployed	14	43.8%
Retired	12	37.5%
Parenthood		
Yes	18	56.3%
No	14	43.8%
Psychiatric family history		
Yes	14	43.8%
No	18	56.2%

SD, standard deviation.

**TABLE 2:** Detailed data about psychiatric disorder and comorbidities.

Current episode duration	17.8 ± 17.0 months	
Mean ± SD	17.8 ± 17.0	
Max	84	
Min	1	
History of previous depressive episodes	N	%
Yes	29	
No	3	
Age of first depressive episode	39.6 ± 14.8 Y.O.	
Mean ± SD	39.6 ± 14.8	
Max	68	
Min	16	
History of suicide attempt		
Yes	26	81,3%
No	8	18.7%
Previous hospitalizations		
Yes	10	31.3%
No	22	68.8%
Bipolar disorder		
MDD	22	68.8%
BD	10	31.3%
Psychotherapy involvement		
Yes	8	25%
No	24	75%
Physical comorbidity		
YES	20	62.5%
NO	12	37.5%
Hypertension	8	25%
Dyslipidaemia	5	15.6%
Oncologic disease	2	6.3%
Cardiovascular disease	7	2.1%
Neurological disease	3	9.4%
Rheumatological disease	0	0%
Gastrointestinal disease	0	0%
Hypothyroidism	3	9.4%
Diabetes	3	9.4%
Respiratory disease	2	6.3%
Psychiatric comorbidity		
Yes	13	40.6%
No	19	29.4%
Psychiatric comorbidity	N	%
OCD	4	12.5%
Panic disorder	6	18.8%
Anxiety disorder	4	12.5%
Nutritional disorder	1	3.1%
Substance use		
No	15	46.9%
Yes	17	53.1%
Benzodiazepines	3	9.4%
Alcohol	3	9.4%
Nicotine	14	43.8%
THC	2	6.3%

SD, standard deviation; MDD, major depressive disorder; BD, bipolar disorder; OCD, obsessive-compulsive disorder; physical comorbidity refers to somatic conditions while psychiatric comorbidity refers to co-occurring psychiatric condition in addition to the primary diagnosis.

**TABLE 3:** Information about esketamine treatment.

Esketamine side effects	N	%
Yes	8	25%
No	24	75%
Nausea	3	9.4%
Incontinence	3	9.4%
Blood pressure increase	3	9.4%
Dissociation	1	3.1%
Psychiatric therapy under esketamine treatment		
Antidepressant	5	15.6%
Antidepressant and antipsychotic	2	6.3%
Antidepressant and stabilizer	14	43.8%
Antidepressant, stabilizer and antipsychotic	11	34.4%
Planned discontinuation for inefficacy^1^		
Yes	10	31.3%
No	22	68.7%
Ongoing therapy^2^		
Yes	13	40.6%
No	19	59.4%
End of treatment		
Yes	19	59.4%
No	13	40.6%
Hospitalization during esketamine treatment		
Yes	2	6.3%
No	30	93.7%
Relapse during esketamine treatment		
Yes	8	25%
No	24	75%
Remission (MADRS)		
Yes	14	43.8%
No	18	56.2%
Response (MADRS)		
Yes	15	46.9%
No	17	53.1%

1 refers to cases in which the treating clinician had decided to discontinue intranasal esketamine due to insufficient clinical benefit, based on routine clinical judgment over the course of treatment.

2 refers to patients who, at the time of data extraction, were still receiving intranasal esketamine within the TRD, treatment program, having not yet completed or discontinued the protocol.

The sample reflects a population with a high burden of disease. Patients had treatment-resistant depression with a mean duration of the current episode of approximately **18 months**, and **81.3%** had a personal history of suicide attempts. The clinical complexity was further compounded by a high prevalence of **physical comorbidities (62.5%)** and **psychiatric comorbidities (40.6%)**. Consistent with the severity of illness in this real-world cohort, **84.4%** of patients were receiving polypharmacotherapy, including combinations of antidepressants, mood stabilizers, and/or antipsychotics, underscoring the refractory nature of their condition.

Baseline and post-treatment symptom severity (at the 12th esketamine administration) were assessed using MADRS, HAM-D, HAM-A, and BPRS scales, with results summarized in Table 4. Patients were categorized as **responders** or **non-responders** based on changes in scale scores. Within-group comparisons between baseline and follow-up showed significant improvement across all psychiatric scales in the total sample and in responders. A graphical representation of treatment response is provided in Supplementary Figure S1.

**TABLE 4:** Scores of psychometric scales at baseline and 2-month follow-up.

​	In total (N = 32)		Responders		Non-responders	
Psychiatric scale	Baseline value	Follow-up	Baseline value	Follow-up	Baseline value	Follow-up
MADRS	25.3	12.9	23.9	4.3	26.5	20.5
HAM-D	18.6	11.0	18.5	4.9	18.6	15.8
HAM-A	15.6	10.7	14.4	4.4	16.1	13.6
BPRS	31.9	25.6	32.7	21.1	31.2	30.2

Responders are defined when the follow-up value decreased at least to 50% of baseline value (MADRS, HAM-D, HAM-A) or ≥20% in BPRS.

### Genotype-phenotype associations and psychometric outcomes

This study investigated genetic variants related to esketamine metabolism and pharmacodynamics. Phenotypic classifications for ***CYP2B6*** *,* ***CYP2C9***, and ***CYP3A4***, as well as genotypes for ***BDNF*** and ***OPRM1***, are reported in Table 5; raw data are available in Supplementary Table S1. Genotype distributions for BDNF and OPRM1 did not deviate from Hardy–Weinberg equilibrium (p > 0.05).

**TABLE 5:** Genotypes of selected genetic variant included in the study.

Pharmacokinetic variants				
​	PM (%)	IM (%)	NM (%)	RM (%)
CYP2B6	3 (9.4%)	11 (34.4%)	12 (37.5%)	6 (18.7%)
CYP2C9	1 (3.1%)	10 (31.3%)	21 (65.6%)	​
CYP3A4	​	3 (9.4%)	29 (90.6%)	​
Pharmacodynamic variants				
​	Val/Val (%)	Val/Met (%)	Met/Met (%)	HWE p-value
*BDNF* (rs6265)	16 (50%)	14 (43.8%)	2 (6.2%)	0.1334
​	A/A (Ref)	A/G	G/G	​
*OPRM1* (rs1799971)	24 (75%)	5 (15.6%)	3 (9.4%)	0.7128

HWE, Hardy-Weinberg equilibrium.

**FIGURE 2:**
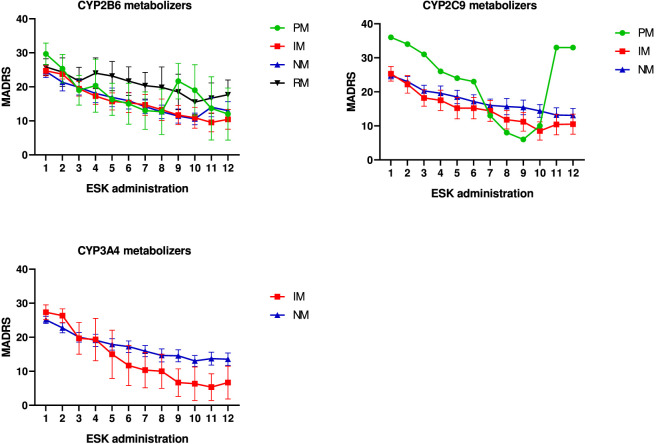
Improvement of MADRS scores following 12 esketamine administrations subdivided according to CYP450 phenotypes of 2B6, 2C9, and 3A4.

No statistically significant differences were found between metabolizer status (CYP2B6, CYP2C9, CYP3A4) and scores on any of the psychiatric rating scales. Notably, one patient classified as a poor metabolizer for CYP2B6 and another for CYP2C9 experienced clinical relapse during the observation period. The trajectory of depressive symptom severity (MADRS scores) in relation to metabolizer status is illustrated in Figure 2.

**FIGURE 3:**
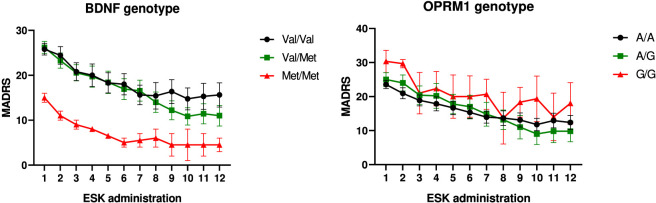
Clinical improvement measured by MADRS scores over 12 esketamine administrations, stratified by pharmacodynamic genotypes. Left: patients stratified by *BDNF rs6265 genotype* (*Val/Val, Val/Met, Met/Met*). Right: patients stratified by *OPRM1 rs1799971 genotype* (A/A, A/G, G/G). Data are presented as mean ± standard error of the mean (SEM). The analysis showed no overall significant association between MADRS score changes and genotypes for either *BDNF* (p = 0.127) or *OPRM1* (p = 0.730).

Regarding ***BDNF* rs6265**, no overall significant association with MADRS scores was observed (p = 0.127). However, pairwise comparisons revealed a significant difference between ***Val/Val*** and ***Met/Met*** genotypes (p = 0.044). As shown in Figure 3, *Met/Met* carriers had significantly lower baseline MADRS scores compared to other genotypes, with differences already evident at the first esketamine administration (*Val/Val* vs *Met/Met*: p = 0.007; *Val/Met* vs *Met/Met*: p = 0.006).

No significant association was found between ***OPRM1* rs1799971** genotypes and MADRS scores (p = 0.730; see Figure 3). Nonetheless, all genotype groups showed significant symptom improvement at the 1-month follow-up (8th administration).

### Haplotype analysis and drug–gene interaction

**FIGURE 4:**
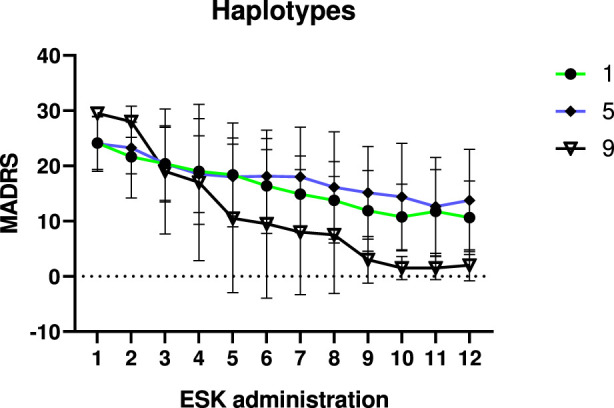
Visualization of 3 haplotypes with the trend towards significant differences between each other. ESK administration was defined as the number of esketamine administrations received by each patient. Values 1, 5, and 9 represent combined metabolic phenotype categories for *CYP3A4*, *CYP2C9*, and *CYP2B6*, defined as follows: 1 = NM/NM/NM; 5 = NM/NM/IM; 9 = IM/IM/IM.

To assess the combined effect of key genes involved in esketamine metabolism, an exploratory haplotype analysis was performed, acknowledging the limited sample size per group. Patients were categorized into **11 haplotype groups** based on their CYP2B6, CYP2C9, and CYP3A4 metabolizer status. No overall significant differences were found between groups (p = 0.575). Haplotype classifications are presented in Table 6, and three groups—**NM-NM-NM**, **NM-NM-IM**, and **IM-IM-IM**—are visualized in Figure 4 due to their trend toward significance.

**TABLE 6:** Haplotype groups of CYP3A4, 2C9, and 2B6.

Haplotype	CYP3A4	CYP2C9	CYP2B6	N
1	NM	NM	NM	8
2	NM	IM	IM	1
3	NM	NM	RM	2
4	IM	NM	RM	1
5	NM	NM	IM	8
6	NM	PM	NM	1
7	NM	IM	NM	3
8	NM	IM	RM	3
9	IM	IM	IM	2
10	NM	NM	PM	2
11	NM	IM	PM	1

When comparing symptom improvement from baseline at 2 weeks, 1 month, and 2 months, the following haplotypes showed significant improvement at the **1-month follow-up**: **NM-NM-NM** (p = 0.027), **NM-PM-NM** (p = 0.037), and **IM-IM-IM** (p = 0.017). At **2 months**, significant improvement was observed in: **NM-NM-NM** (p = 0.001), **NM-NM-IM** (p = 0.016), **IM-IM-IM** (p = 0.001), and **NM-IM-PM** (p = 0.021).

### Drug-drug interactions

Based on the literature and patients’ medication list, fluvoxamine, a moderate inhibitor of CYP2C9 and CYP3A4 (Marzolini et al., 2022), was found in 5 patients. No other drugs in the cohort were considered relevant inhibitors or inducers of CYP450 enzymes that could affect esketamine metabolism.

**FIGURE 5:**
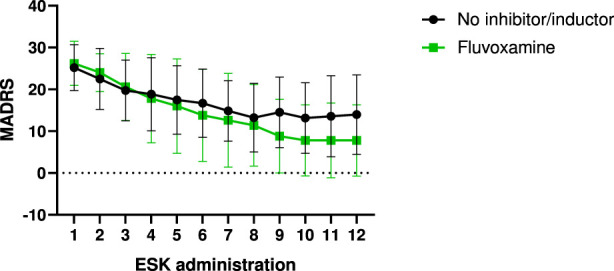
Clinical improvement measured as MADRS scores divided according to the presence of fluvoxamine in the treatment. ESK administration was defined as the number of esketamine administrations received by each patient.

Although statistical analysis did not reveal a significant association - likely due to the small sample size (N = 5) - patients receiving fluvoxamine showed a trend toward greater clinical improvement (Figure 5). Notably, 3 of these 5 patients were carriers of reduced-function alleles for *CYP2C9* and/or *CYP3A4*. This suggests a possible drug–gene interaction, where fluvoxamine-induced enzymatic inhibition may have led to **phenoconversion** from a normal or intermediate metabolizer genotype to a functional poor metabolizer phenotype. This could have increased esketamine exposure and contributed to enhanced therapeutic response. Importantly, no adverse effects related to esketamine were observed in patients receiving fluvoxamine. Although exploratory, these findings support the need for future studies to stratify patients not only by genotype but also by concurrent use of CYP450 inhibitors or inducers.

### Adverse effects of esketamine treatment

Among the 32 patients included in the final analysis, 25% experienced adverse drug reactions (ADRs) related to esketamine treatment. Reported ADRs included nausea, blood pressure increase, urinary incontinence, and dissociation, as detailed in Table 3.

No significant associations were found between the occurrence of ADRs and any specific **CYP450 genotype**: *CYP2B6*: p = 0.3515; *CYP2C9*: p = 0.7856; *CYP3A4*: p = 0.2935. Similarly, no significant associations were observed for the **pharmacodynamic variants**: *BDNF* (rs6265): p = 0.2397; *OPRM1* (rs1799971): p = 0.8653.

Although gender was not significantly associated with ADR risk, there was a trend toward higher incidence in females (p = 0.0614), warranting further investigation in larger samples.

### Dosage of esketamine

**FIGURE 6:**
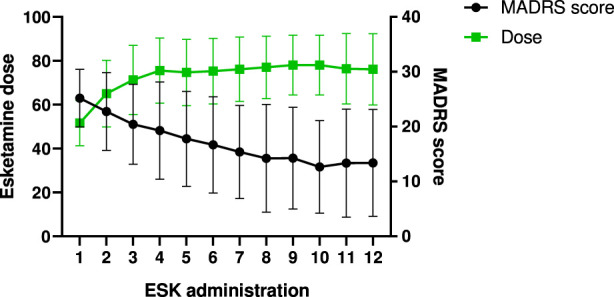
Correlation between esketamine doses and MADRS score during 12 administrations. ESK administration was defined as the number of esketamine administrations received by each patient.

Esketamine was administered according to the established clinical protocol. The initial dose was 56 mg, except for patients aged ≥65 years, who started at 28 mg. Subsequent dose adjustments were made based on individual efficacy and tolerability. The doses administered at the 12th treatment session are reported in Table 7. No significant association was found between esketamine dose at 2 months and improvement in MADRS scores (p = 0.1774). A graphical representation of this analysis is provided in Figure 6.

**TABLE 7:** Esketamine doses at the 12th administration.

Dose	% of patients (N)
28 mg	6.3 (2)
56 mg	15.6 (5)
84 mg	78.1 (25)

### Other variables and treatment effect

We have not found any significant association between psychiatric rating scores following esketamine administration and any of the clinical and genetic variable.

Subsequently, we analysed the association between selected variables and remission status. A significant association was found between remission and concurrent psychotherapy. Full results for all tested variables are presented in Table 8.

**TABLE 8:** Selected variables and their association with remission on esketamine (ordered according to their association with remission status).

Variable	p-value	Effect size	95% CI	Test
**Psychotherapy**	**0.0002**	OR = ∞	4.01 to ∞	χ^2^
Occupation status (working, not working, retired)	0.0676	-	-	χ^2^
*BDNF (rs6265)* genotype	0.1489	-	-	χ^2^
Organic pathologies	0.1977	OR = 0.38	0.08 to 1.51	Spearman
Duration of episode	0.4718	r = −0.13	−0.47 to 0.24	Spearman
Age of onset of first episode	0.6291	r = −0.09	−0.43 to 0.28	Spearman
Unipolar or bipolar depression	0.6309	OR = 0.69	0.14 to 3.30	χ^2^
Familiarity of psychiatric disorders	0.6434	OR = 0.72	0.21 to 2.94	χ^2^
Substance use	0.6879	OR = 0.75	0.20 to 2.82	χ^2^
Gender	0.8206	OR = 0.85	0.23 to 3.26	χ^2^
Psychiatric comorbidity	0.8336	-	-	χ^2^
Age	0.8673	r = −0.03	−0.38 to 0.33	Spearman
CYP2B6 metabolizer status	0.9034	-	-	χ^2^
Educational level	0.9644	-	-	χ^2^

95% CI = 95% confidence interval, OR = odds ratio, χ^2^ - Chi-square, r = Spearman rho. Categorical associations were assessed using chi-square tests; odds ratios and 95% confidence intervals are reported for 2 × 2 comparisons. Odds ratios are oriented so that the outcome event = remission. OR values of ∞ occur when no non-remitters are observed in the exposed group. Spearman rank correlation coefficients (r, rho) are reported for associations between continuous variables, along with exact 95% confidence intervals and corresponding p-values.

Although age and gender were not statistically significant predictors of treatment response, patients of younger age groups (18–34 years) tended to show lower scores on psychiatric rating scales (Supplementary Figure S2). Additionally, **female patients** exhibited higher average MADRS scores throughout the treatment period (Supplementary Figure S3).

**FIGURE 7:**
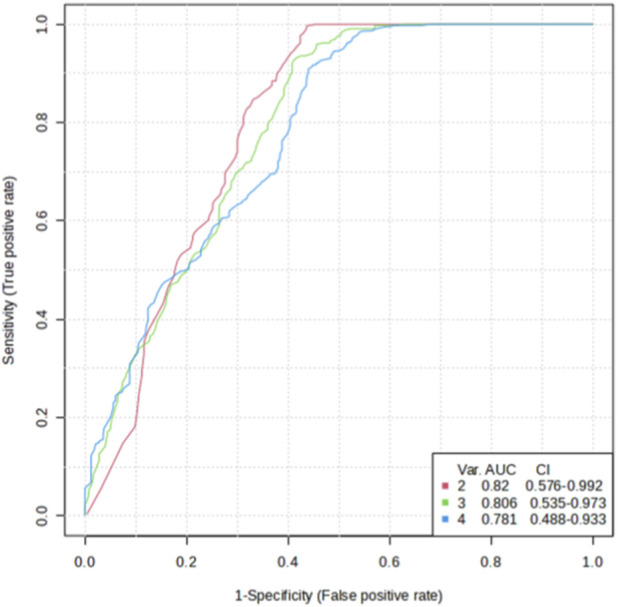
Discrimination power of various variables on remission on esketamine treatment (2 variables = psychotherapy + *BDNF* rs6265 genotype, 3 variables = + CYP2B6 metabolizer status, 4 variables = + occupation).

To evaluate the predictive value of selected variables for remission, a receiver operating characteristic (ROC) curve was constructed (Figure 7). The combination of psychotherapy status and BDNF genotype yielded the highest discriminative power, with an **Area Under the Curve (AUC) of 0.82**. However, the 95% confidence interval (0.576–0.992) was wide, reflecting the uncertainty due to the small sample size.

In summary, in this naturalistic TRD cohort, intranasal esketamine was associated with significant improvements across all psychometric scales, with 43.8% of patients achieving remission after 12 administrations. No meaningful dose–response relationship emerged, and commonly explored clinical, demographic, and pharmacogenetic variables showed limited predictive value. Psychotherapy involvement was the only factor associated with remission. Overall, these findings highlight a robust clinical effect of esketamine while suggesting that most stratification variables provide minimal contribution to outcome prediction in real-world TRD.

## Discussion

In this real-world TRD cohort, intranasal esketamine led to a consistent improvement across all symptom domains, with nearly half of the patients achieving remission after 12 administrations and a safety profile aligned with previous clinical experience. Notably, no significant associations emerged between treatment outcomes and the majority of demographic, clinical, or pharmacogenetic variables examined, and no clear dose–response pattern was observed. Psychotherapy involvement was the only factor meaningfully associated with remission, whereas commonly proposed predictors—such as metabolizer status, *BDNF Val66Met*, or *OPRM1* variants—did not demonstrate substantial clinical utility in this sample. These findings highlight both the effectiveness of esketamine in routine clinical practice and the limited predictive contribution of many variables frequently explored in ketamine research.

In our TRD cohort, the mean baseline MADRS score 25.3, indicating moderate depression severity, while average HAM-A scores reflected mild anxiety. Most participants were women, with the highest prevalence in the 45–65 age range (50%) and all were of Caucasian origin, characteristics consistent with previous TRD studies (Liu et al., 2021). Over the 2-month treatment, all the patients significantly improved their depression as well as anxiety symptoms, although not all patients achieved full response or remission. Out of 32 TRD patients included, 14 (43.8%) reached remission at the 2-month follow-up. This rate aligns with findings from a multicenter real-world study reporting 40.6% remission at 3 months (Martinotti et al., 2022). Longer-term data indicate that sustained esketamine treatment may enhance outcomes: in the REAL-ESK study, 76.2% of patients who continued therapy for 6 months were responders or remitters, and among the 15 non-responders at 6 months, four achieved significant improvement by 12 months (Rosso et al., 2025). In our sample, 25% experienced adverse effects—primarily nausea, transient blood pressure elevations, and urinary incontinence, while dissociative symptoms were reported by only one patient.

In our study, esketamine efficacy did not differ significantly between sexes. Notably, mean MADRS scores remained higher in female patients throughout the treatment period, which likely reflects baseline severity rather than a differential treatment effect. We examined employment status, educational level, current episode duration, and psychiatric comorbidity, but found no significant differences between remitters and non-remitters. Consistent with a meta-analysis comparing ketamine/esketamine efficacy in unipolar *versus* bipolar depression (Medeiros et al., 2022), we observed no difference in response between these diagnostic groups. We also analysed the effect of age of depression onset and family history of psychiatric disorders, but neither showed an association with response to esketamine treatment. These findings suggest that commonly cited non-specific predictors (Medeiros et al., 2024) may have limited utility in real-world TRD populations.

We observed a strong association between adjunctive psychotherapy and remission with esketamine. While our finding refers specifically to remission, previous studies have described improvements in symptom severity and durability of response when esketamine or ketamine was combined with psychotherapeutic interventions: a recent case series reported promising outcomes in patients with TRD and PTSD treated with esketamine alongside psychotherapy (Roullet et al., 2025), and an open-label study suggested that cognitive behavioral therapy may prolong the antidepressant effects of intravenous ketamine in severely ill patients (Wilkinson et al., 2017). In contrast, esketamine dose was not associated with response in our study. This finding diverges from randomized controlled trials and pharmacokinetic analyses (Perez-Ruixo et al., 2021; Su et al., 2017). These results underscore the potential role of combined treatment strategies while questioning the clinical relevance of dose escalation in esketamine therapy. Optimizing esketamine treatment in combination with psychotherapy may require moving beyond a single dose-escalation model toward a multidimensional framework, in which pharmacologic exposure and psychotherapeutic intensity are jointly adjusted.

Our hypothesis that slower metabolism would lead to higher esketamine exposure and greater symptom improvement was not supported. Although patients classified as slower metabolizers of CYP2B6, CYP2C9, and CYP3A4 showed numerically greater improvement in MADRS scores, these differences did not reach statistical significance. Similarly, concomitant use of CYP2C9 and CYP3A4 inhibitors, such as fluvoxamine, was not associated with a statistically significant advantage.

In this real-world TRD cohort, no distinct clinical, demographic, or genetic profile emerged as a reliable predictor of response beyond compliance with established TRD criteria. The absence of a narrow responder phenotype aligns with current evidence showing that esketamine responders remain highly heterogeneous across clinical trials and observational studies. Our data therefore suggest that, in practice, patient selection should follow standard TRD diagnostic and eligibility criteria as defined in the product label, rather than relying on additional stratifying features.

In routine clinical practice, dosing flexibility with intranasal esketamine is limited: the approved titration schedule generally leads most patients to reach the 84-mg dose early in treatment, a pattern fully reflected in our cohort. This is consistent with recent Phase 4 and real-world data showing that the 84-mg dose is the one most commonly adopted in clinical settings and may be associated with equal or potentially greater clinical benefit without added safety burden, while earlier studies (e.g., TRANSFORM-1, Fedgchin et al., 2019) did not always detect a clear dose–response gradient. Within these protocol-driven constraints, our findings indicate that nominal dosing contributes only marginally to inter-individual variability. Factors such as metabolic phenotype, concomitant pharmacotherapy, and engagement in psychotherapy are likely to be more influential determinants of treatment outcome.

This study is limited by its small sample size, short follow-up, clinical heterogeneity, and absence of placebo control. Nevertheless, within this real-world cohort, clinical, demographic, and genetic variables did not meaningfully differentiate responders from non-responders.

Direct evaluation is needed to determine whether psychotherapy reliably enhances remission and durability of response in patients treated with esketamine. The interaction between esketamine-induced neuroplasticity and therapeutic engagement may represent a clinically modifiable dimension of outcome rather than a fixed patient characteristic. Future research should therefore focus on pragmatic, prospective, multicenter real-world cohorts examining the combined optimization of esketamine dosing strategies and structured psychotherapeutic support.

## Conclusions

In this real-world TRD cohort, no clear demographic, clinical, or genetic profile reliably predicted response to intranasal esketamine. Adjunctive psychotherapy was the only factor associated with remission. However, due to the limited sample size, the study may have lacked sufficient statistical power to detect smaller effect sizes. Taken together, these findings, rather than supporting a narrow biological responder phenotype, point toward a multidimensional model of treatment optimization, in which pharmacologic exposure and psychotherapeutic engagement are co-determined in clinical practice. Future research should aim to confirm these findings in larger, prospective cohorts.

## Data Availability

The original contributions presented in the study are included in the article/Supplementary Material, further inquiries can be directed to the corresponding author.
