# Supplementary material for: Pilot study on esketamine response in treatment-resistant depression: impact of pharmacogenetic, clinical, and demographic variables
Source: Front Pharmacol. 2026 May 5;17:1783538. doi: 10.3389/fphar.2026.1783538 (PMC13185135; doi:10.3389/fphar.2026.1783538)
Supplement: Supplementary file 1 [file DataSheet1.pdf]

Supplementary Table S1: Genotypes and phenotypes of the studied genes/genetic variants in our study participants

ID of patient	**CYP3A4 Genotype**	**CYP3A4 Phenotype**	**CYP2C9 Genotype**	**CYP2C9 Phenotype**	**CYP2B6 Genotype**	**CYP2B6 Phenotype**	**BDNF (rs6265)**	**OPRM1 (rs1799971)**
1	*1/*1	NM	*1/*1	NM	*1/*1	NM	Met/Met	A/A
2	*1/*1	NM	*1/*2	IM	*1/*6	IM	Val/Val	A/G
3	*1/*2	NM	*1/*0	NM	*1/*22	RM	Val/Val	A/A
4	*1/*22	IM	*1/*1	NM	*5/*22	RM	Val/Met	A/A
5	*1/*1	NM	*1/*1	NM	*4/*9	IM	Val/Val	A/A
6	*1/*1	NM	*2/*3	PM	*1/*1	NM	Val/Val	A/A
7	*1/*1	NM	*1/*2	IM	*1/*5	NM	Val/Val	A/A
8	*1/*1	NM	*1/*1	NM	*1/*1	NM	Val/Met	A/A
9	*1/*1	NM	*1/*1	NM	*1/*6	IM	Val/Val	A/A
10	*1/*1	NM	*1/*1	NM	*5/*5	NM	Val/Met	A/A
11	*1/*2	NM	*1/*1	NM	*4/*6	IM	Val/Val	A/A
12	*1/*1	NM	*1/*1	NM	*1/*1	NM	Val/Met	A/A
13	*1/*1	NM	*1/*1	NM	*1/*1	NM	Val/Val	A/A
14	*1/*1	NM	*1/*2	IM	*1/*5	NM	Met/Met	A/A
15	*1/*1	NM	*1/*1	NM	*1/*6	IM	Val/Val	G/G
16	*1/*1	NM	*1/*1	NM	*1/*1	NM	Val/Met	A/A
17	*1/*1	NM	*1/*1	NM	*1/*6	IM	Val/Val	A/A
18	*1/*1	NM	*1/*3	IM	*1/*4	RM	Val/Met	A/A
19	*1/*22	IM	*1/*2	IM	*1/*2	IM	Val/Met	A/A
20	*1/*1	NM	*1/*1	NM	*4/*5	RM^	Val/Met	A/A
21	*1/*1	NM	*1/*1	NM	*6/*6	PM	Val/Val	A/A
22	*1/*1	NM	*1/*1	NM	*1/*6	IM	Val/Met	A/A
23	*1/*22	IM	*1/*2	IM	*1/*6	IM	Val/Val	A/G
24	*1/*1	NM	*1/*2	IM	*1/*22	RM	Val/Met	G/G
25	*1/*1	NM	*1/*2	IM	*1/*4	RM	Val/Val	A/G
26	*1/*1	NM	*1/*1	NM	*1/*6	IM	Val/Met	A/A
27	*1/*1	NM	*1/*1	NM	*1/*1	NM	Val/Met	A/G
28	*1/*1	NM	*1/*1	NM	*6/*6	PM	Val/Val	A/A
29	*1/*1	NM	*1/*1	NM	*1/*6	IM	Val/Val	A/A
30	*1/*1	NM	*1/*2	IM	*1/*1	NM	Val/Met	A/G
31	*1/*1	NM	*1/*2	IM	*6/*6	PM	Val/Met	G/G
32	*1/*1	NM	*1/*1	NM	*1/*1	NM	Val/Val	A/A

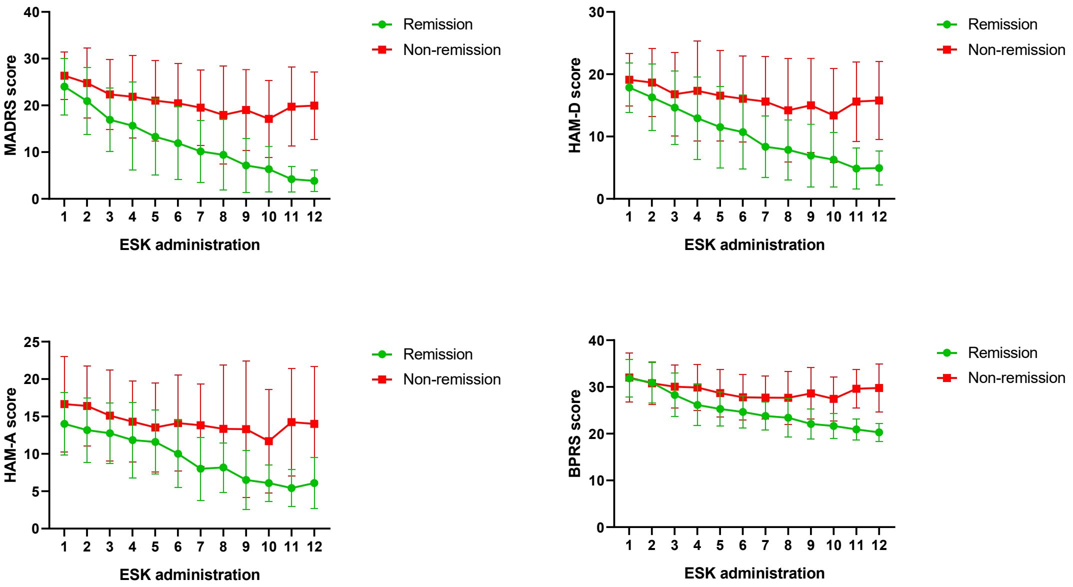

Supplementary Figure S1: Scores of psychiatric rating scales following 12 esketamine administrations divided in responders and non-responders

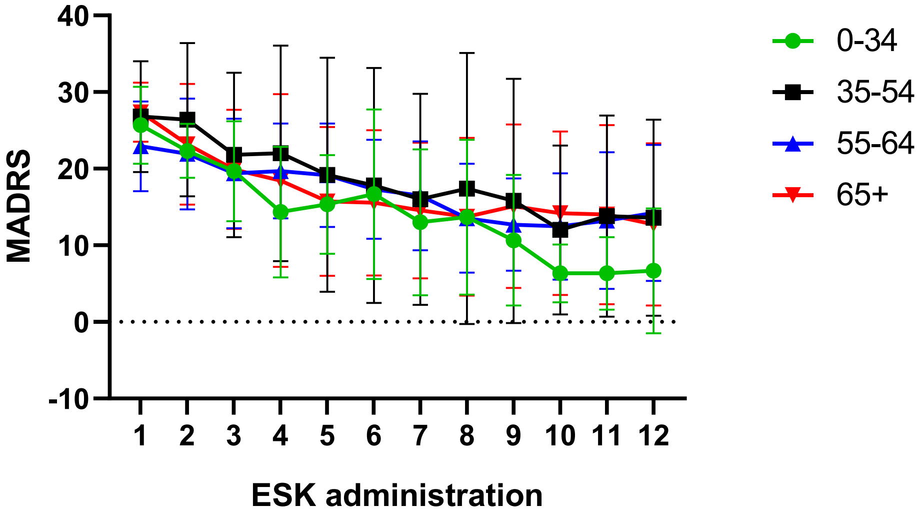

Supplementary Figure S2: Differences in MADRS score during 2-month esketamine treatment divided into age groups

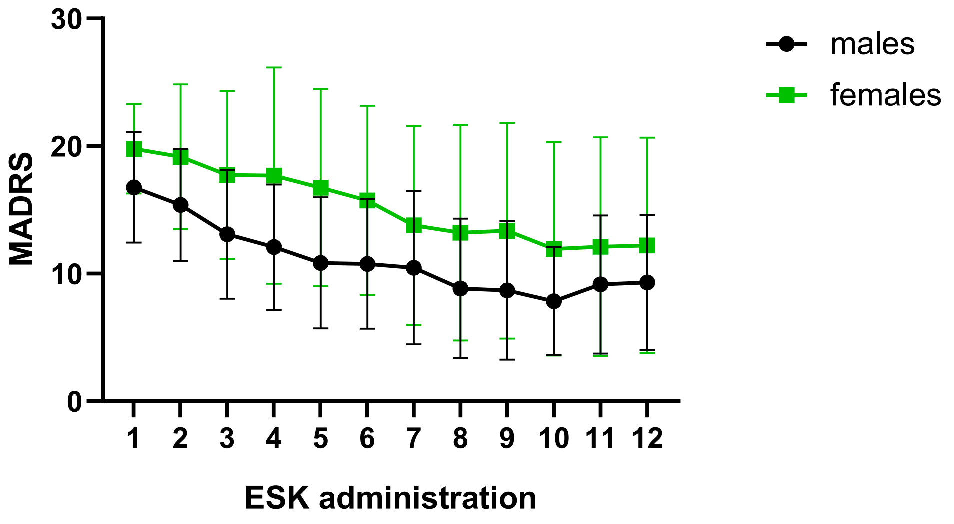

Supplementary Figure S3: Gender differences in MADRS score during 12 esketamine administrations
